# Noncontiguous Genome Sequence of *Mycobacterium septicum* Strain DSM 44393^T^

**DOI:** 10.1128/genomeA.00574-13

**Published:** 2013-08-15

**Authors:** Mohamed Sassi, Catherine Robert, Didier Raoult, Michel Drancourt

**Affiliations:** Unité de Recherche sur les Maladies Infectieuses et Tropicales Emergentes (URMITE), UMR CNRS 7278, IRD 198, INSERM 1095, Faculté des Médicine, Marseille, France

## Abstract

The rapidly growing *Mycobacterium septicum* rarely causes pulmonary infections. We report here the draft genome sequence of *M. septicum* strain DSM 44393^T^, isolated from catheter-related bacteremia and initially identified as a member of *Mycobacterium fortuitum.*

## GENOME ANNOUNCEMENT

The rapidly growing *Mycobacterium septicum* is found in water environments (1, 2). *M. septicum* rarely causes pulmonary infections (3, 4) but the name was coined after its initial isolation from the blood of a patient with catheter-related bacteremia, when it was identified as a member of the *Mycobacterium fortuitum* group (5). In order to get further knowledge on this group of mycobacteria, we report here the first draft genome sequence of *M. septicum* strain DSM 44393^T^.

The *M. septicum* genome was sequenced using the 454 GS FLX Titanium pyrosequencing system (Roche, Boulogne-Billancourt, France). The 454 sequencing generated 371,276 reads (142,575,954 bp), assembled into contigs and scaffolds using Newbler version 2.6 (Roche) and checked using CLC Genomics Workbench v 4.7.2 (CLC bio, Aarhus, Denmark). Functional annotation was achieved using Prodigal (6) and BLASTp searches against the National Center for Biotechnology Information (NCBI) nonredundant (NR), UniProt (http://www.uniprot.org/), and COG databases (7). tRNA and rRNA genes were predicted using Aragorn and RNAmmer, respectively (8, 9).

*M. septicum* strain DSM 44393^T^ comprises 173 contigs (including 159 contigs of >1,500 bp) in 7 scaffolds. The draft genome size is 6,879,294 bp, with 66.73% G+C content. There are 56 RNAs. Of the 6,748 predicted genes, 6,692 (92.63%) are protein-coding genes, including 4,752 (71.01%) genes assigned to a putative function, 1,381 genes (20.64%) annotated as hypothetical proteins, and 199 (2.97%) genes identified as ORFans. We found 178 genes encoding resistance to aminoglycosides, β-lactamases, fosfomycin, fucidic acid, fluoroquinolones, macrolide-lincosamide-streptogramin B, phenicol, rifampin, tetracycline, trimethoprim, and glycopeptides. A total of 119 type VII secretion system proteins were annotated by comparison with reannotated *Mycobacterium tuberculosis* strain H37Rv. The *M. septicum* genome encodes 487 prophages and phage proteins and 11 clustered regularly interspaced short palindromic repeats (CRISPRs), according to the fast phage search tool (Phast) (10) and the CRISPERfinder online software program (http://crispr.u-psud.fr/Server/).

*M. fortuitum* members were determined to be the closest species to *M. septicum* based on the 16S rRNA gene sequence similarity. Accordingly, *M. septicum* presents 83.32% nucleotide sequence identity of core proteins with *M. fortuitum* subsp. *fortuitum*, confirming that these are two distinct species within the same group of mycobacteria (11). We identified 5,977 orthologous genes shared between *M. fortuitum* subsp. *fortuitum* and *M. septicum*. Also, 715 genes present in *M. septicum* are not found in *M. fortuitum*. These data could serve to set up new molecular tools for the refined identification of *M. septicum* in both environmental and clinical specimens to identify precise epidemiological and clinical features associated with this emerging opportunistic pathogen.

### Nucleotide sequence accession number.

This whole-genome shotgun project has been deposited at DDBJ/EMBL/GenBank under the accession no. CBMO00000000.1. The version described in this paper is the first version.
